# Identification of vagal afferent nerve endings in the mouse colon and their spatial relationship with enterochromaffin cells

**DOI:** 10.1007/s00441-024-03879-6

**Published:** 2024-02-22

**Authors:** Nick J. Spencer, Melinda A. Kyloh, Lee Travis, Timothy J. Hibberd

**Affiliations:** Visceral Neurophysiology Laboratory, Flinders Health and Medical Research Institute & College of Medicine and Public Health, Flinders University of South Australia, GPO Box 2100, Bedford Park, Adelaide, South Australia 5042 Australia

**Keywords:** Gut-brain axis, Enterochromaffin cell, Serotonin, Spinal afferent, Vagal afferent anterograde tracing

## Abstract

Understanding how the gut communicates with the brain, via sensory nerves, is of significant interest to medical science. Enteroendocrine cells (EEC) that line the mucosa of the gastrointestinal tract release neurochemicals, including the largest quantity of 5-hydroxytryptamine (5-HT). How the release of substances, like 5-HT, from enterochromaffin (EC) cells activates vagal afferent nerve endings is unresolved. We performed anterograde labelling from nodose ganglia in vivo and identified vagal afferent axons and nerve endings in the mucosa of whole-mount full-length preparations of mouse colon. We then determined the spatial relationship between mucosal-projecting vagal afferent nerve endings and EC cells in situ using 3D imaging. The mean distances between vagal afferent nerve endings in the mucosa, or nearest varicosities along vagal afferent axon branches, and the nearest EC cell were 29.6 ± 19.2 μm (*n* = 107, *N* = 6) and 25.7 ± 15.2 μm (*n* = 119, *N* = 6), respectively. No vagal afferent endings made close contacts with EC cells. The distances between EC cells and vagal afferent endings are many hundreds of times greater than known distances between pre- and post-synaptic membranes (typically 10–20 nm) that underlie synaptic transmission in vertebrates. The absence of any close physical contacts between 5-HT-containing EC cells and vagal afferent nerve endings in the mucosa leads to the inescapable conclusion that the mechanism by which 5-HT release from ECs in the colonic mucosa occurs in a paracrine fashion, to activate vagal afferents.

## Introduction

The vagus nerve is crucial in the gut-brain axis, influencing fluid and food intake (Borgmann et al. 2021), heart rate, energy metabolism, respiration, and gut motility (Breit et al. 2018). Additionally, its role in managing anxiety and depression is gaining interest (Breit et al. 2018; Margolis et al. 2021).

The axons that make up the vagus nerve are predominately sensory in origin that extensively innervate much of the gastrointestinal tract (Powley 2000, Zagorodnyuk et al. 2001; Powley et al. 2013). Progress in understanding vagal afferent innervation along the gut has been significant (Page 2023), but their activation by hormones from mucosal enteroendocrine cells (EEC) is not fully understood. Enterochromaffin (EC) cells are the most abundant type of EEC (Erspamer 1957; Martin et al. 2017; Koo et al. 2021; Song et al. 2023) and synthesize high quantities of 5-hydroxytryptamine (5-HT; serotonin) (Treichel et al. 2018; Spencer and Keating 2022). They are mechanically sensitive and release 5-HT in response to distension or contraction of the gut wall (Bertrand 2006, Alcaino et al. 2018; Keating and Spencer 2010; Treichel et al. 2018; Spencer and Keating 2022). Studies have suggested that communication between EEC and vagal afferent nerve endings involves fast synaptic transmission (Kaelberer et al. 2018). Indeed, evidence has been provided that close contacts occur between EEC and neurons in culture dishes, but strong evidence of synapses in situ is lacking. Recent anterograde tracing studies (Dodds et al. 2022) found no evidence of close contacts between EC cells and spinal afferent endings in the mucosa, contradicting earlier theories of synaptic activation by 5-HT release from EC cells (Bellono et al. 2017).

Here, we conducted unilateral anterograde tracing from mouse nodose ganglia in vivo, identifying colonic vagal afferent axons and nerve endings for the first time. We quantified their proximity to mucosal 5-HT containing EC cells and observed no close contacts. This suggests that substances from 5-HT-containing EC cells act on neighboring vagal afferents in a paracrine fashion through diffusion.

## Methods

### Nodose injections of anterograde tracer in vivo

**Fig. 1:**
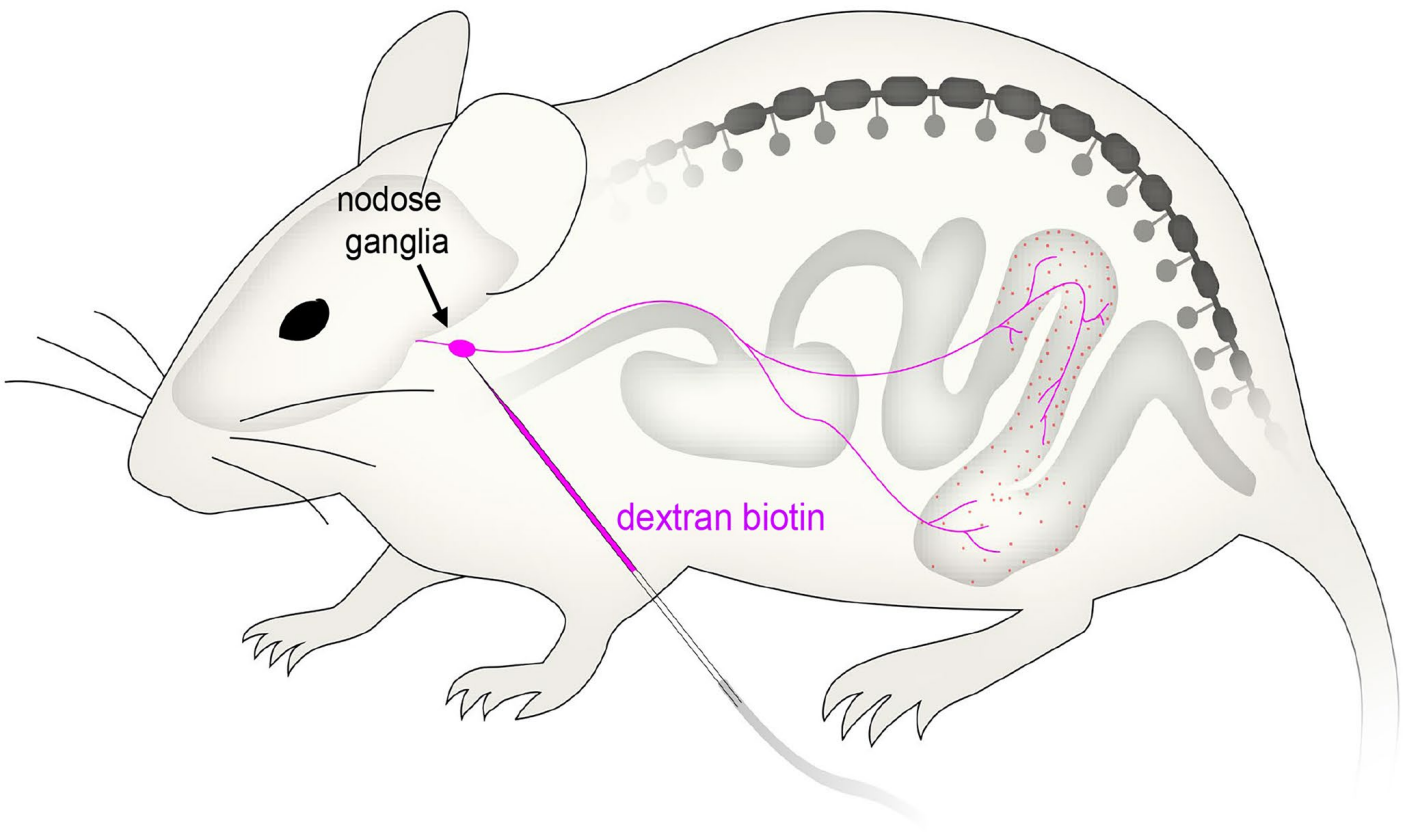
Diagrammatic representation of the fine glass micropipette used to inject minute quantities of dextran biotin into a single nodose ganglion of anaesthetized mice

Male and female C57BL/6 mice (20–25 g) were anesthetized by isoflurane inhalation (2–3% in 1 L/min O_2_) and an incision ~ 10–15 mm made in the neck. The right nodose ganglion was exposed, and dextran biotin (20%, 0.5–1.0 μL; #D1956, Molecular Probes, Eugene, OR, USA) was injected via glass micropipette (inner diameter: ~ 5–10 μm; #TW150-4, World Precision Instruments, Sarasota, FL, USA; Fig. 1) using a custom-made nitrogen-driven spritz system (10–15 psi, 5 pulses of 1-s duration at 0.3 Hz; Biomedical Engineering, Flinders University). Skeletal muscle and skin were sutured after injection. Animals had 7–10 days to recover before euthanasia by exsanguination under deep inhaled isoflurane anesthesia. We define the distal colon and rectum as the region that spans 30 mm from the anal sphincter. This includes the region of the colon above and below the pelvic bone. The proximal colon we defined as the region within 20 mm of the ceco-colonic junction which encompasses the distinct haustral folds. The mid colon is the region between these two proximal and distal defined regions.

### Immunohistochemistry

The colon was removed, opened along the mesentery, and pinned mucosal side up in PBS (0.1 M). It was fixed overnight in 4% paraformaldehyde, cleared with dimethyl sulfoxide, and blocked for 1 h with 10% normal horse serum (Life Technologies Gibco™ #16050–122). Preparations were immersed for 4 h in Cy3-conjugated streptavidin (9 µg/mL; Jackson ImmunoResearch #016–160-084) and then incubated in CGRP (rabbit anti-CGRP; 1:2000; Peninsula Laboratories #T-4032) and 5-HT (goat anti-5-HT; 1:2000; ImmunoStar #20079) antibodies for two nights. Preparations were incubated for 4 h in secondary antibodies (donkey anti-goat Cy5 and donkey anti-rabbit FITC; both 7.5 µg/mL; Jackson ImmunoResearch #705–175-147 and #711–095-152), mounted on slides with carbonate-buffered glycerol (pH 8.6). All solutions were diluted in PBS with 0.1% sodium azide, and PBS washes were conducted between antibody steps.

### Image acquisition and analysis

Preparations were initially observed with an Olympus IX71 epifluorescence microscope using appropriate discriminating filters and imaged at × 4 to × 40 magnification using a CoolSNAP™ camera and AnalySIS Image 5.0 software. Vagal afferent metrics were measured using ImageJ 1.52p software. Preparations were then examined at × 20 magnification on a Zeiss LSM880 confocal microscope. Images were acquired with Imaris × 64 version 8.4.1 software, adjusting gain and offset for optimal quality. Final digital composites consisted of 0.53–1 µm z-slices, covering the full depth of the colon mucosa. Distances between mucosal vagal afferent structures and EC cells were measured in Imaris × 64 version 8.4.1 software. Shortest distances were identified in 3D rotated images. Lengths were calculated with Imaris’ *measurement points* feature, calibrated based on image magnification.

### Morphological classification

Vagal afferent nerve endings in the mucosa were classified using a method similar to our previous work on colon-projecting spinal afferents (Spencer et al. 2014). “Simple endings” feature a single, unbranched axon with few varicosities. “Complex-type” endings have multiple varicose axons branching from one parent axon, without preferential directionality. The third type, “lamellar-type” endings, resembles the rectal intraganglionic lamellar endings (rIGLEs) in mouse colon (Spencer et al. 2014).

### Experimental design and statistical analysis

Data are presented as mean ± standard deviation. *N* indicates number of animals, and *n* is the number of observations. Statistics were generated with Prism 8 (GraphPad Software), considering *p* < 0.05 significant. Distances between vagal afferent nerve endings or varicosities and EC cells were analyzed using the two-way Mann–Whitney tests.

## Results

### Characteristics of vagal afferent labelling in mouse colon

Colonic vagal afferent innervation appeared sparse, as labelled axons were found in less than half of the animals tested. This was not due to incomplete axon filling, as labelled nerve endings were consistently bright and distinct. In colons without labelling, tracer uptake in the nodose ganglion was confirmed by labelled axons in the esophagus, stomach, or small intestine. Of 24 mice injected, 10 animals showed anterogradely labelled axons and nerve endings as far as the colon. In 6 of these 10 animals, nerve endings were identified in the mucosa. Vagal afferent axons were observed in the proximal colon and to a lesser extent in the mid colon, but never in the distal colon or rectum (region within 30 mm from the anal sphincter).

**Fig. 2:**
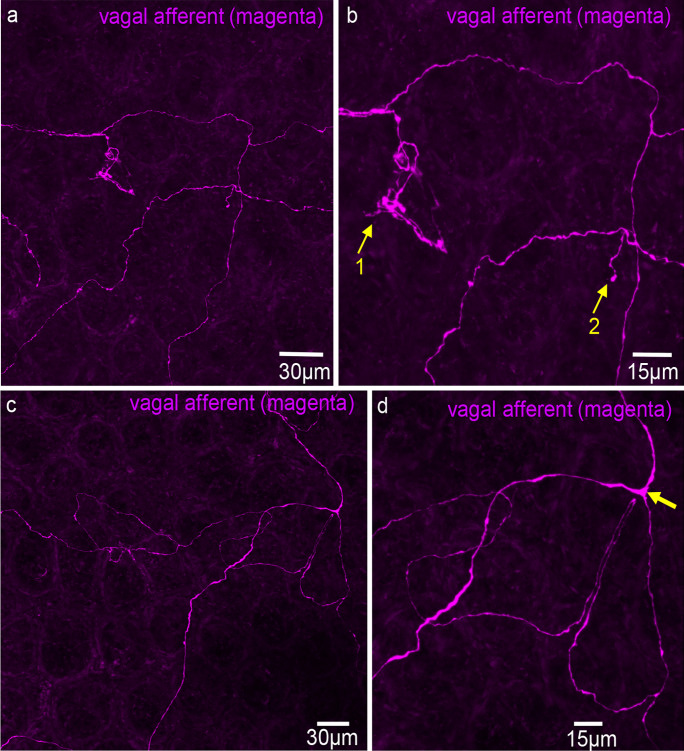
Anterogradely labelled vagal afferent axons in the mid colon. **a** Single fine vagal axons course over the mucosa surface, with no preferential direction that aligns with the underlying crypts. Two different types of ending are shown in **a**, a complex- and simple-type ending. **b** An enlarged image from panel **a**. The complex ending is indicated by arrow 1 and the simple ending by arrow 2. **c** Vagal afferent axons traversing through the mucosa in no preferential orientation to the crypts. **d** Image in **c** is shown on expanded scale. Note that the diameter of axon changes substantially, especially at the bifurcation point (see arrow)

**Fig. 3:**
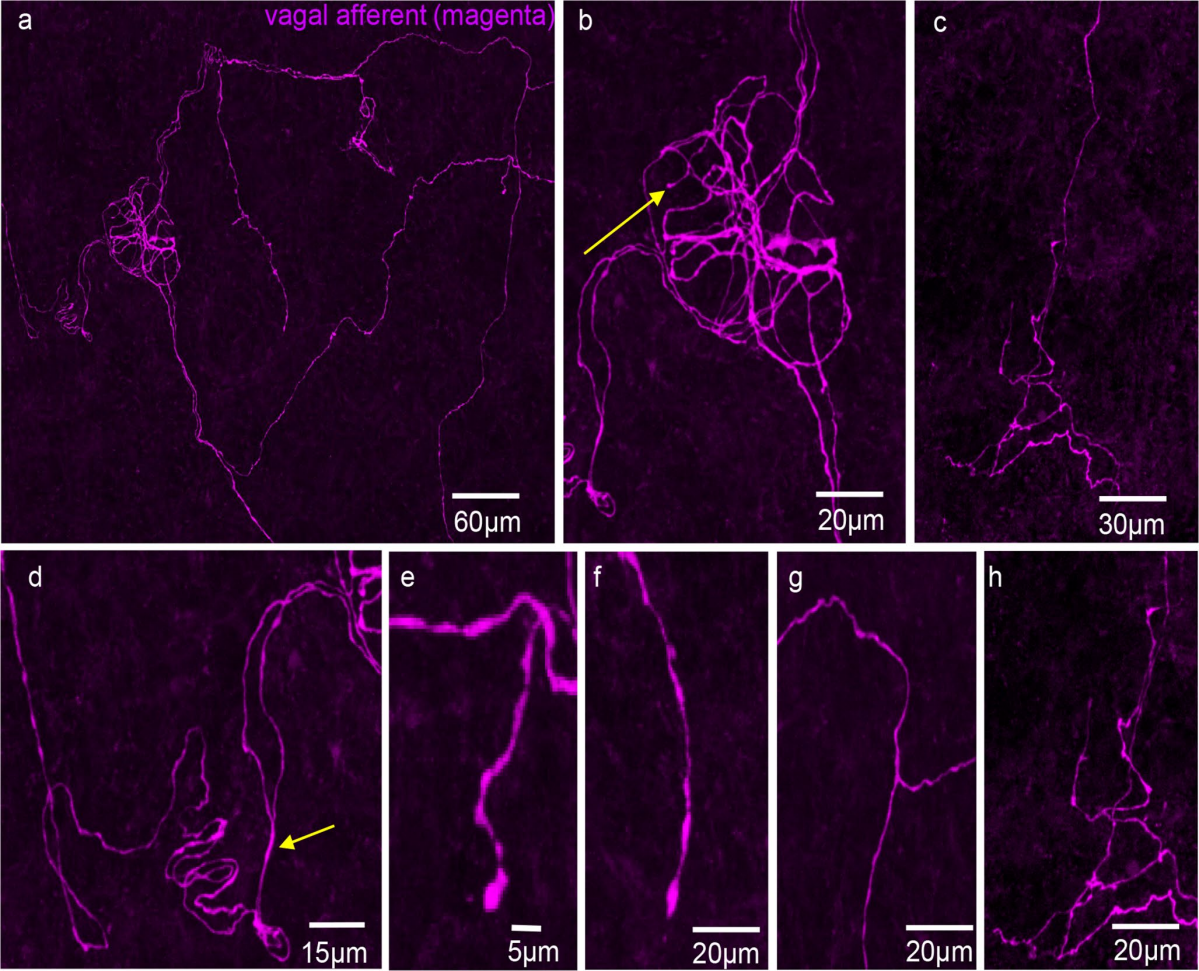
**a** Anterogradely labelled vagal afferent axons and nerve endings in the proximal-mid colon. **b** This image is an expanded region shown in panel **a**. Dense innervation is shown around a submucosal ganglion. The arrow shows a simple-type ending in the submucosal ganglion. **c** A complex-type vagal afferent ending in the mucosa. Image taken from a different mouse to panels **a** and **b**. Note that the single axon gives rise to multiple fine axon terminals with few varicosities. The axons do not align in any preferential direction. **d** An expanded region from panel **a**, where a single vagal axon branches into two axons that follow a similar trajectory (see arrow). Note that the diameter of the axon is thicker before the axon bifurcation occurs. **e** A simple-type ending from panel **a**. **f** Another simple-type ending from panel **a**. **g** A bifurcation of a single axons branching into two axons. **h** An expanded region from the complex ending in panel **c**

**Fig. 4:**
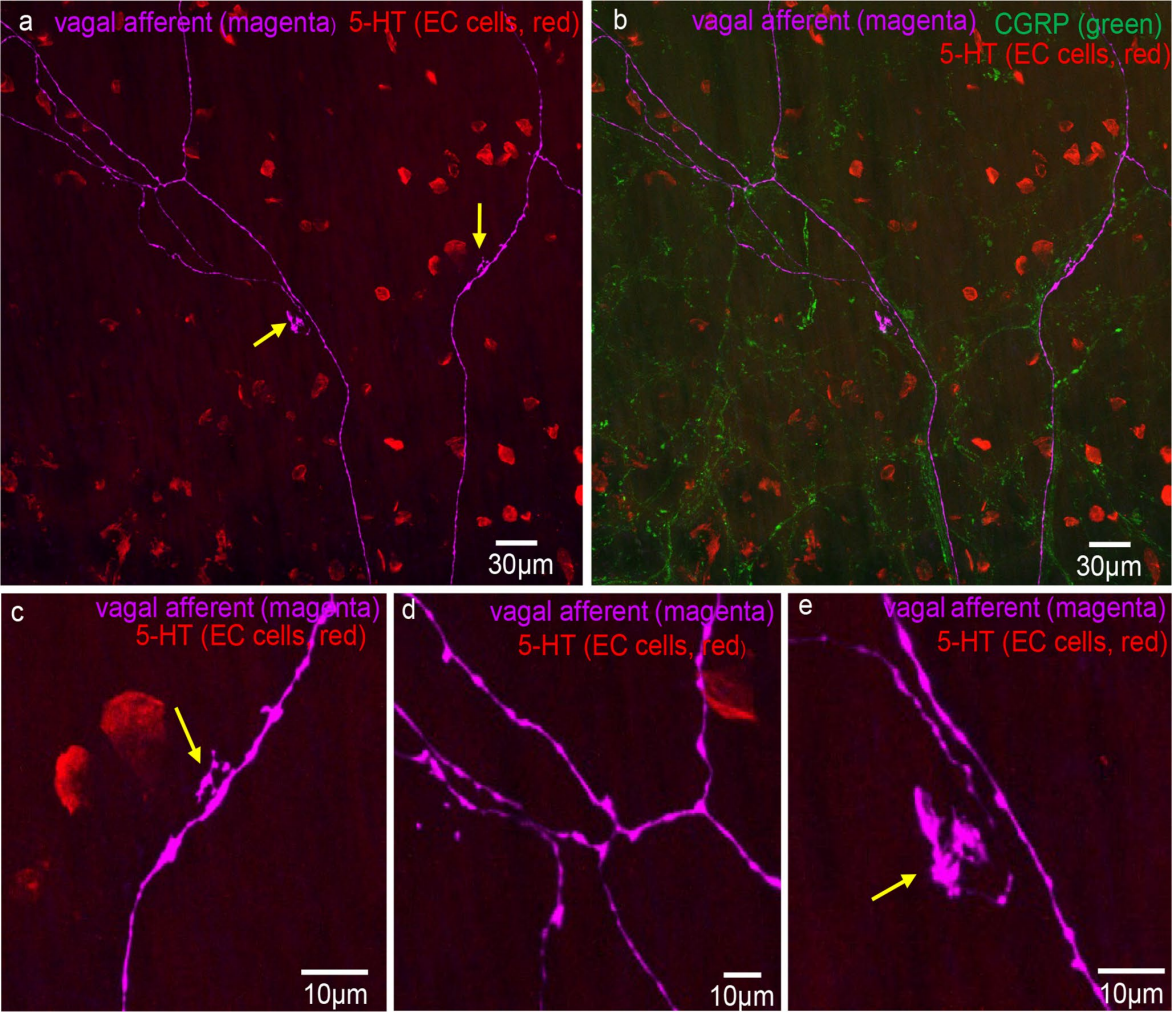
Confocal micrograph showing vagal afferent axons and endings with 5-HT staining in red (EC cells). Two lamellar-type endings are shown indicated by the two arrows. **b** The image in **a** with CGRP immunoreactivity (green) also now superimposed. An outline of the colonic crypts is apparent. **c** The lamellar-type ending that branches off the main vagal axon. This ending is indicated by the right-hand arrow in panel **a**. **d** The bifurcation of vagal axons from the region in the top left hand region of panel **a**. **e** The lamellar-type ending in the mucosa on expanded scale. The ending is indicated by the left-hand arrow in panel **a**

**Fig. 5:**
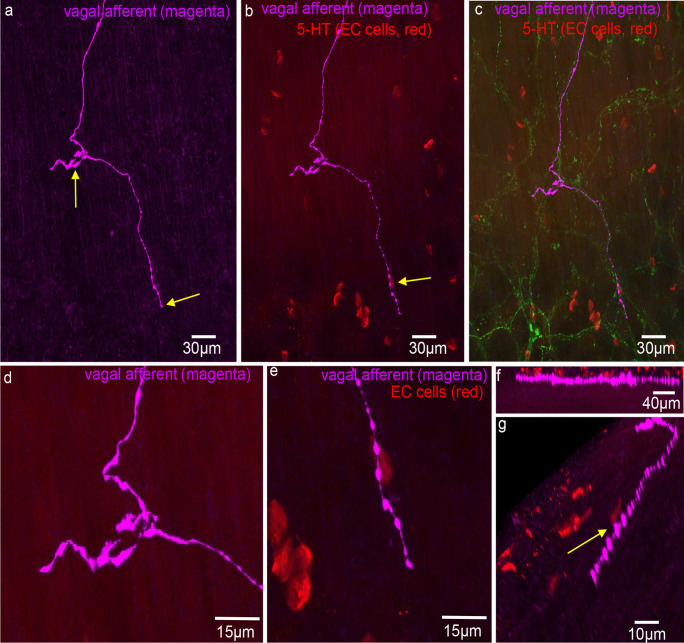
Confocal micrograph showing two different morphological types of vagal afferent ending arising from the same vagal afferent axon. **a** A simple-type vagal afferent ending in the lower (bottom) part of the image, while a lamellar-type ending with flattened lamellar endings is shown toward the middle of the image. These images were taken from the mid colon. **b** 5-HT stain superimposed on panel **a** to reveal the location of EC cells. Note that both nerve endings are not aligned physically close to any EC cells. The arrow shows an EC cell that appears close to a vagal axon of passage that it passes over the EC cell. **c** CGRP (green) stain superimposed on panel **b**. **d** An expanded image of one of the nerve endings in panel **a**. The lamellar morphology is apparent, in contrast to the simple varicose terminal at the lower segment of the image. **e** An expanded region of the image in panel **b**. The varicose vagal axon passes over the EC cell. **f** A side on image (confocal stack) where the vagal axon traverses parallel to the population of EC cells. **g** A rotated image from panel **e**. The arrow shows the varicose axon lying close to the EC cell, but the axon extends past the EC without making any preferential termination with the EC cell

Vagal afferent axons did not preferentially target the myenteric plexus, unlike spinal afferents in mouse colon (Spencer et al. 2020a, b,). They also lacked clear directionality in the submucosa before projecting into the mucosa (Figs. 2 and 3). Three morphological types of endings were identified: simple, complex, and lamellar. Complex endings, with bifurcated axons forming non-directional terminals, and simple endings are both shown in Fig. 2b (arrows 1 and 2). Simple-type endings were also observed in submucosal ganglia (Fig. 3a, b). Lamellar endings, resembling flattened leaf-like structures, are depicted in Fig. 4a, c, and d. In some cases, lamellar- and simple-type endings had the same parent axon (Fig. 5a). Unlike spinal afferent nerve endings, which extensively weave around colonic crypts, vagal afferents did not show similar weaving around these crypts. Additionally, all vagal afferent axons and endings were not immunoreactive to CGRP antibodies, suggesting that they are non-peptidergic (*N* = 10).

### Characteristics of vagal axons and their nerve endings

The diameter of vagal axons varied considerably as they coursed through the submucosa. The mean diameter was 1.19 ± 0.08 µm (Table 1; *n* = 31, *N* = 6). However, diameters varied considerably from 0.46 to 2.3 µm (Table 1; *n* = 31, *N* = 6). The diameter of a single axon was typically thicker before bifurcating into two branches, as illustrated in Fig. 3d (see arrow).

**Table 1:** Characteristics of vagal afferent endings in the mucosa

	**Nerve terminal (diam.) (µm)**	**Axon diameter (µm)**	**Varicosity diam. (major) (µm)**	**Varicosity diam. (minor) (µm)**
Mean	1.19	1.15	2.62	1.71
S.E.M	0.08	0.05	0.07	0.06
S.D	0.42	0.48	0.72	0.65
Min	0.46	0.46	1.0	0.61
Max	2.3	2.4	4.1	3.8
*n* (observations)	31	100	111	110

Thirty-one mucosal-projecting vagal afferent nerve endings were identified and analyzed in detail (*N* = 6). On average, each randomly chosen field of view (FOV) contained 4.5 ± 0.4 axons (range 1–9) and 58.4 ± 5.7 EC cells (range 26–129; *N* = 6). In total, 105 labelled axons and 1343 EC cells were present in all FOVs.

### Spatial relationship between mucosal vagal afferent nerve endings and 5-HT EC cells

**Fig. 6:**
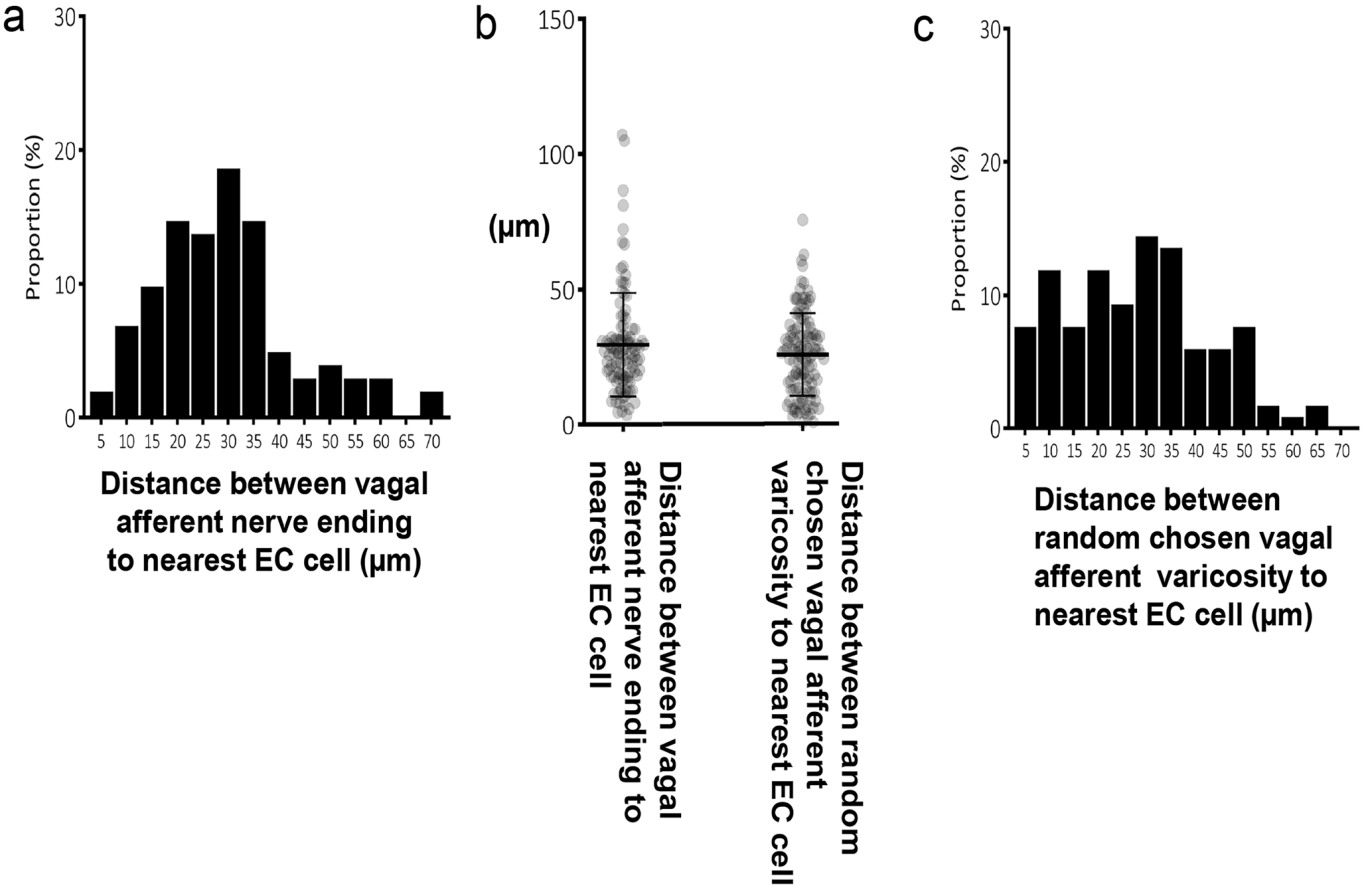
**a** Graph represents the proportion of vagal afferent endings (terminals) to the nearest 5-HT-containing EC cell. **b** The shortest distance between vagal afferent terminal endings to 5-HT-containing EC cells. **c** The proportion of data with the shortest distance between random vagal afferent varicosity to nearest 5-HT-containing EC cell

**Fig. 7:**
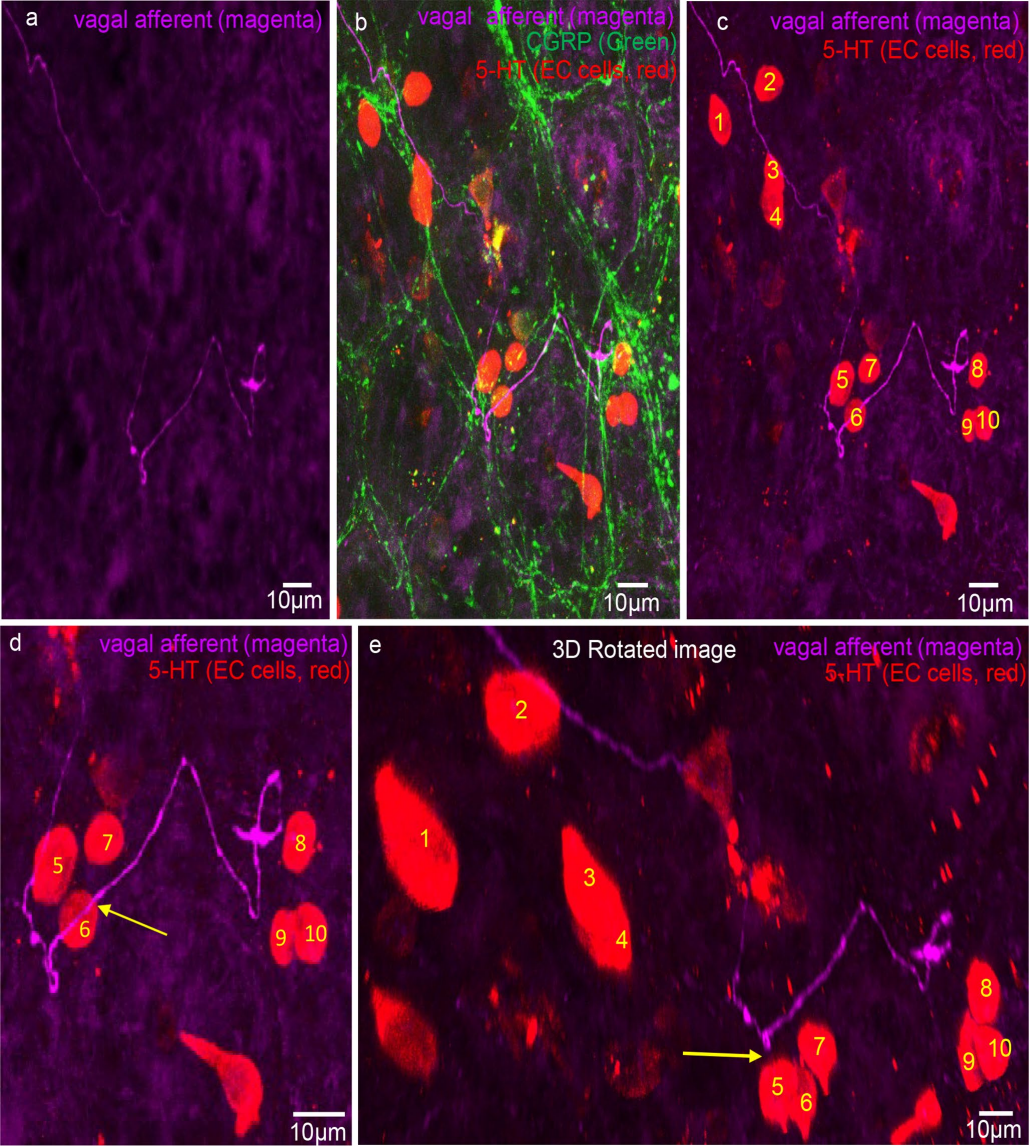
**a** Confocal micrograph showing a simple-type vagal afferent axon and ending in the mucosa. **b** CGRP (green) immunoreactivity superimposed on 5-HT stain (red) for EC cells and magenta showing vagal afferent axon and ending. The honeycomb outline of the colonic crypts can be seen in green which labels peptidergic axons. **c** 5-HT (red) and anterograde-labelled axon (magenta). The numbers indicate individual EC cells. The axon traverses past EC cell number 5 and 6. **d** An expanded segment from panel **c**. **e** A rotated image from panel **d**. The arrow indicates that EC cell numbers 5 and 6 do not touch the vagal axon

**Fig. 8:**
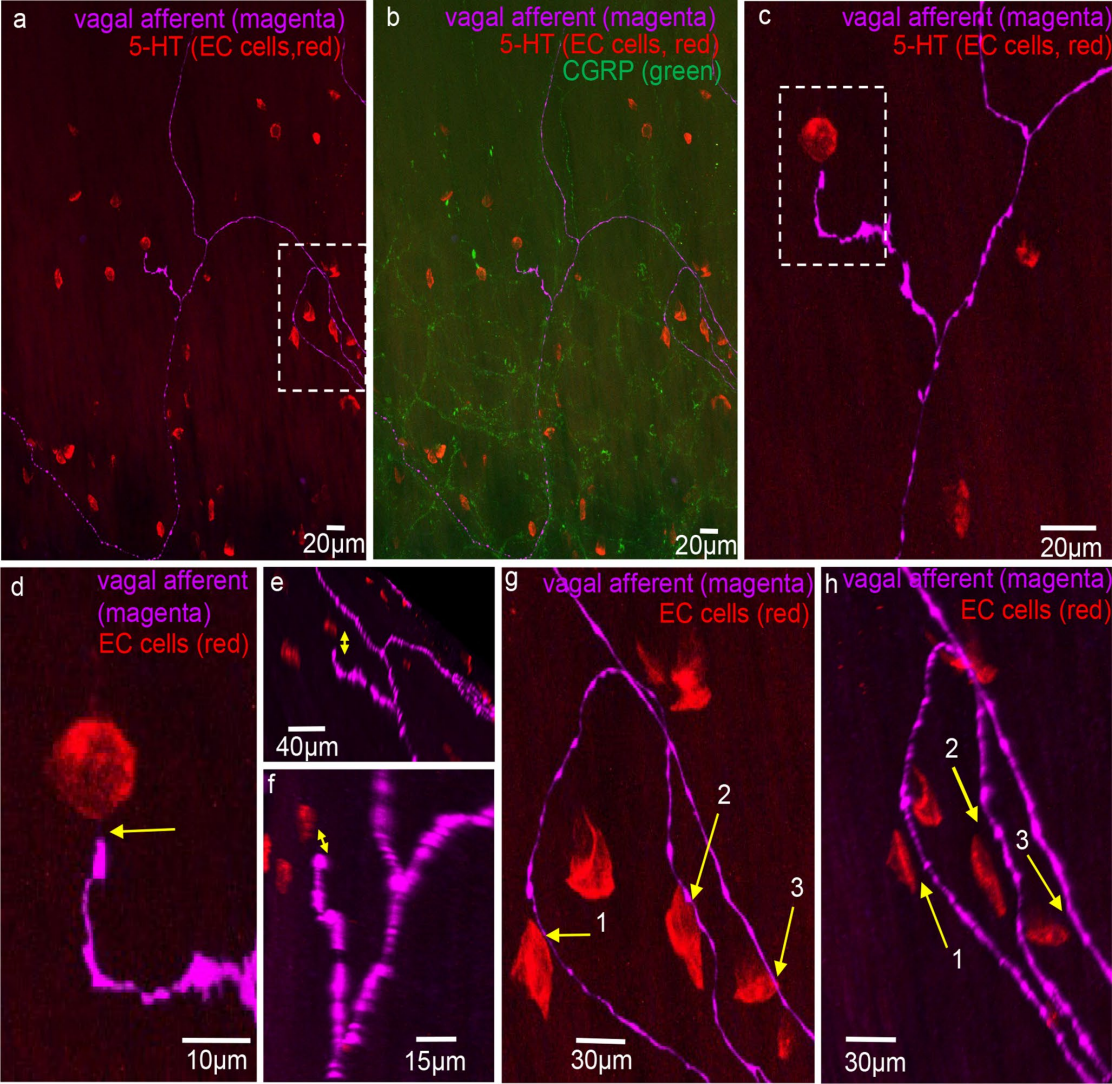
**a** Anterogradely labelled vagal axons (magenta) and 5-HT (red) labelling EC cells in the mucosa of mid colon. The region highlighted by the dotted white box is shown expanded in panel **g**. **b** CGRP (green) labelling superimposed on 5-HT (red, EC cells) and vagal axons (magenta). **c** Vagal axons and simple ending with an EC cell (see dotted white box). **d** Expanded region from panel **c**. The arrow indicates about 3 microns separates the EC cell and the vagal ending. **e** A rotated image of panel **d**. The arrow indicates at least 5 microns separates the EC cell and vagal afferent ending. **f** Another 3D rotated image from panel **d**. Again, many microns separate the EC cell and the vagal axon. **g** The region in panel **a** in the white box. Arrows 1, 2, and 3 show potential close contacts with axons of passage between EC cells and vagal axons of passage. **h** A 3D rotation of image in panel **g**. Arrows 1, 2, and 3 in panel **h** show clear separation of many microns between the vagal axons of passage and same EC cells represented by arrows 1, 2, and 3 in panel **g**

**Fig. 9:**
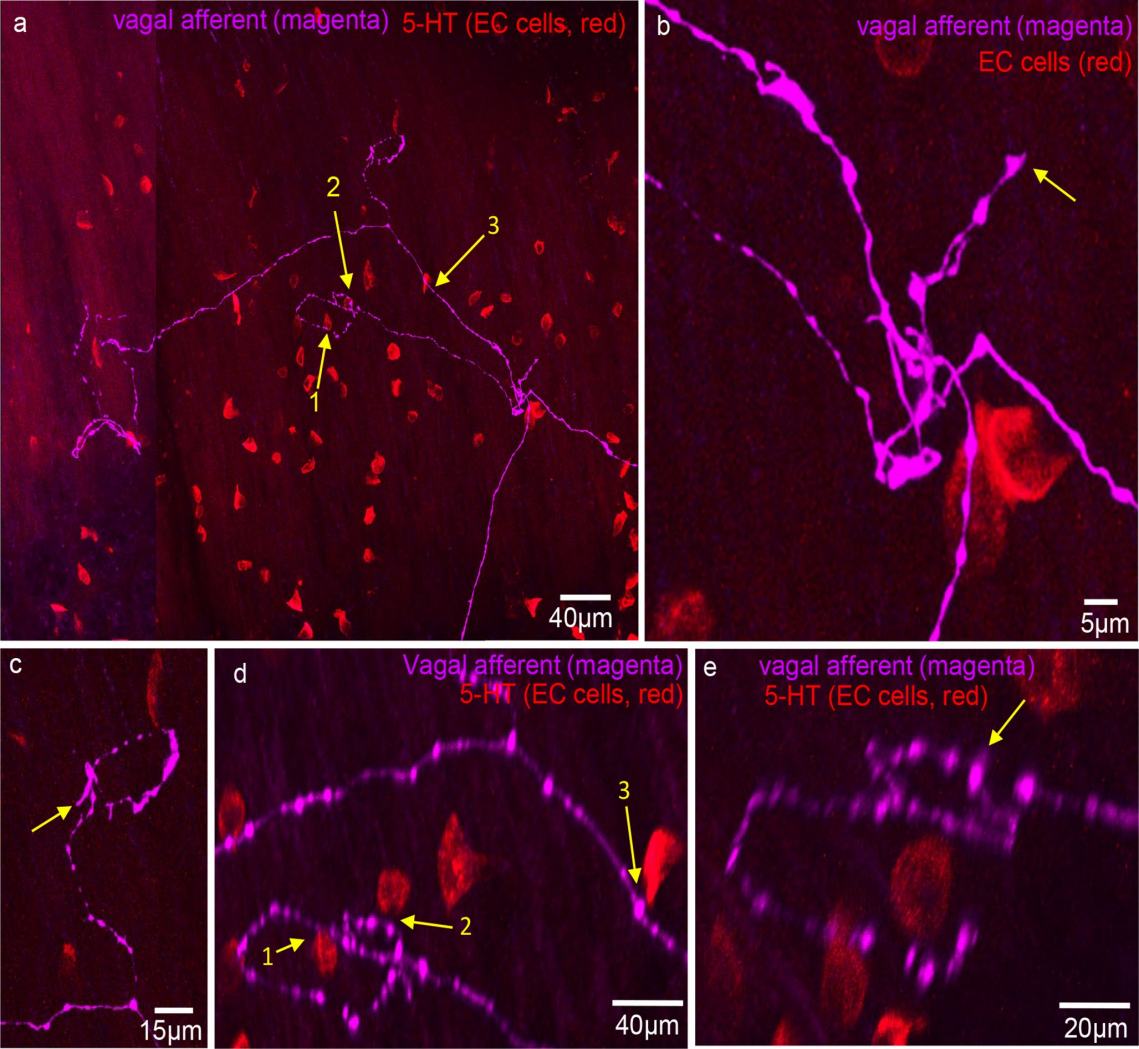
Confocal micrograph showing anterogradely labelled vagal axons and endings (magenta) in the mucosa of mid colon with 5-HT immunohistochemistry (red) showing distribution of EC cells. None of the vagal afferent endings make close contacts with any EC cells. Arrows 1, 2, and 3 show three possible contacts of EC cells with vagal axons of passage. These images are rotated to show an absence of contact between axon and EC cells (see arrows 1–3 in panel **d**). **b** An expanded image from bifurcation of vagal axons shown in panel **a**. The arrow indicates a vagal ending that is not associated with any closely apposed EC cell (i.e., within synapse distance). **d** Rotated image of arrows 1, 2, and 3 in panel **a**. In rotated images, it is clear the axons to passage do not form physical contact with EC cells. **e** Expanded image region from panel **d**. The spaces between EC cells and vagal axons are in the order of microns away

**Fig. 10:**
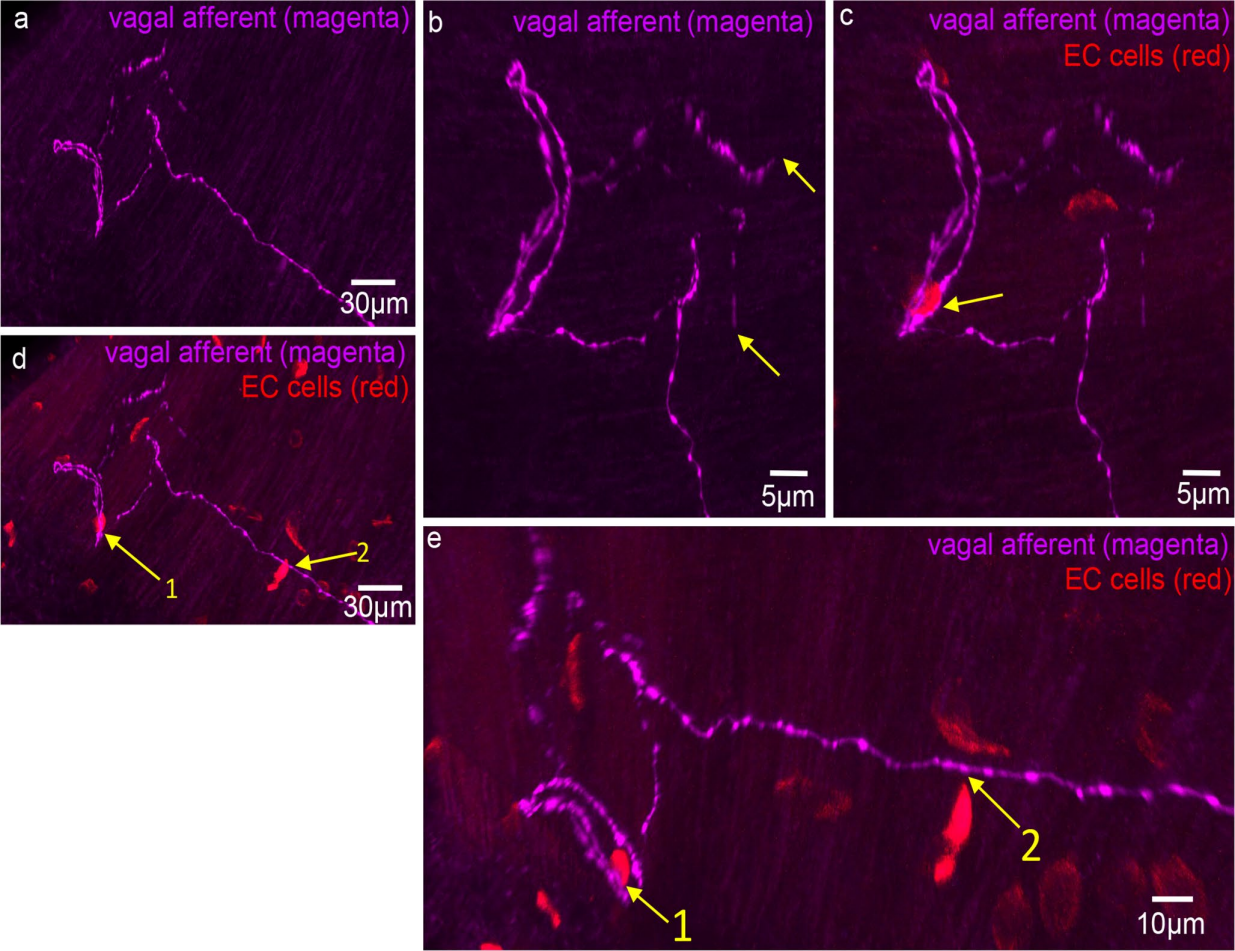
Anterogradely labelled vagal axon and endings in the mucosa of mid colon. This image is shown on expanded scale and shown in the left-hand side of **a**. **b** Expanded scale on the endings in **a**. The arrows indicate the vagal endings. **c** Vagal axons and ending (magenta) and 5-HT-containing EC cells (red). The arrow indicates an EC cell (red) that is close to vagal axons of passage. **d** Increased length of vagal axon where the two arrows indicate close spatial associations between axons of passage and EC cells. **e** A 3D rotated image of **d**. Arrow 2 in fact shows that vagal axon does not come into close contact with the EC cell and is many microns away. Arrow 1 shows that the EC cell and vagal axon of passage are closely apposed, while the vagal ending is not close to any EC cells

Of the 31 vagal afferent nerve endings in the mucosa, the distance to the nearest EC cell was 29.6 ± 19.2 μm (Fig. 6; *n* = 107, *N* = 6). No vagal afferent nerve endings formed close spatial associations with EC cells. In addition, randomly selected varicosities along vagal axons were 25.7 ± 15.2 μm (*n* = 119; *N* = 6) to the nearest EC cell (see Fig. 6). Figures 7, 8, 9, and 10 depict how 3D rotation of images reveals axons several microns from EC cells that initially appeared to form close associations when viewed in a single plane.

In 7 of 1343 EC cells in 34 FOVs, we found a labelled vagal axon of passage, but not nerve endings, in close association with an EC cell. This suggests that close associations occur rarely and not at nerve endings.

## Discussion

We identified vagal afferent nerve endings in the mouse colon mucosa and analyzed their spatial relationship with EC cells in whole-mount preparations. The key finding indicates an absence of close contacts between 5-HT-containing EC cells and mucosal vagal afferent endings, suggesting communication is likely to be paracrine rather than synaptic.

### Characteristics of vagal afferent nerve endings in the mucosa

**Fig. 11:**
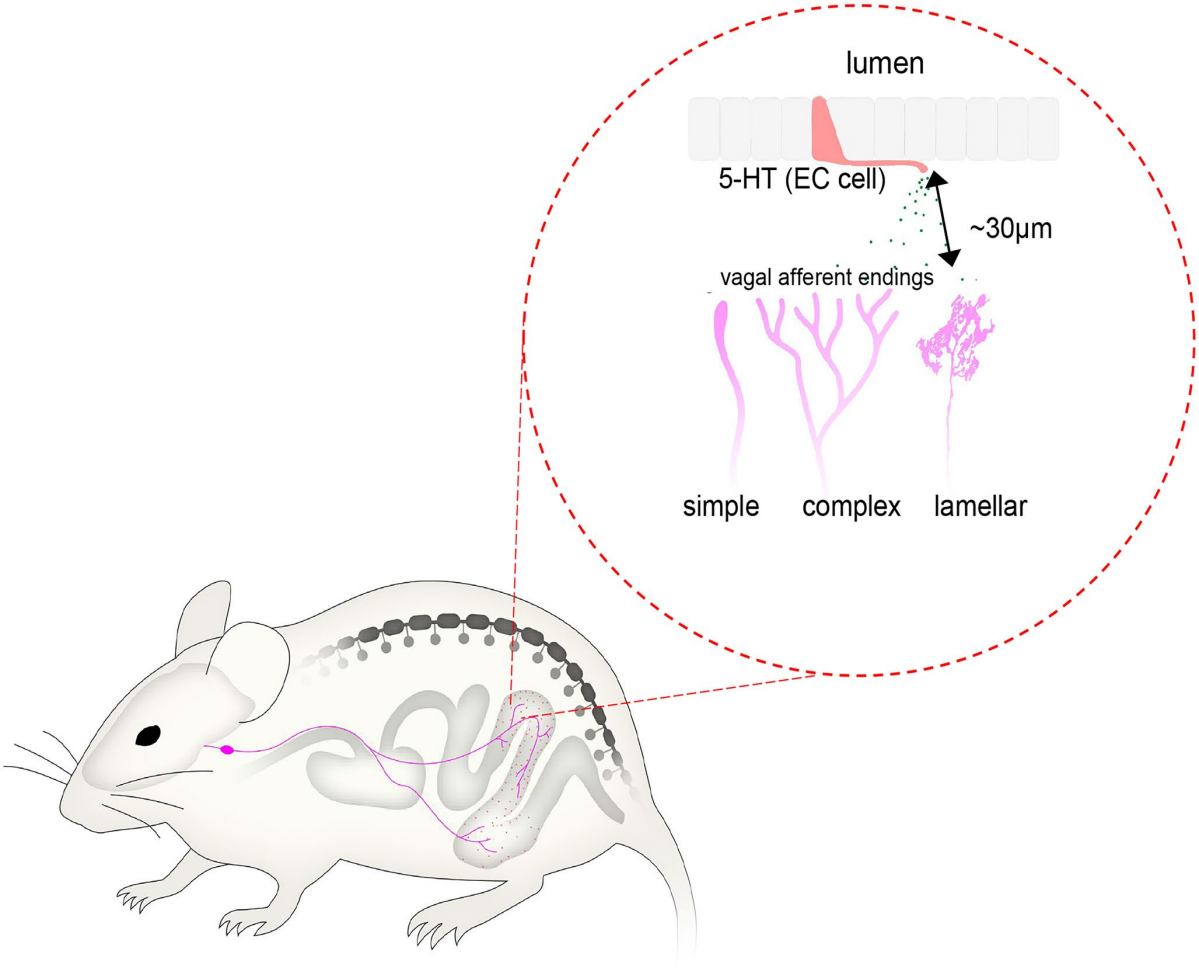
Diagrammatic representation of the three major morphological types of vagal endings identified in the mucosa, consisting of simple, branched, and lamellar-type endings

Vagal afferent endings in the mucosa of the proximal and mid colon of mice display greater morphological variety than spinal afferent endings in the mucosa of the distal colon (Spencer et al. 2014). Three types of vagal afferent endings—simple, complex, and lamellar—were identified in the mucosa of proximal and mid colon (Fig. 11). Simple endings are bare axons with few varicosities and no multiple bifurcations. Complex types have single axons branching multiple times, with non-parallel terminal alignments to neighboring axons (Fig. 3f). Lamellar-type endings comprised flattened, leaf-like endings, analogous to rectal IGLEs identified previously. Indeed, Powley and colleagues reported in the rat and mouse upper small intestine and antrum that vagal afferent collaterals can develop lamellar processes in the mucosa (Powley et al. 2011). In contrast, only one major type of mucosal-projecting spinal afferents was identified previously in the mouse colon that were simple-type endings, consisting of bare endings that lack prominent varicosities along their axons (Spencer et al. 2014). Our findings agree with the conclusions of Powley et al. where they suggested vagal afferents “…did not typically divide or give off terminal spurs in their trajectories through smooth muscle, myenteric plexus, and submucosa…” (Powley et al. 2011). Our data indicate that vagal afferents do not preferentially pass through myenteric ganglia en route to their targets, contrasting with spinal afferents in the mouse colon, which often weave through multiple myenteric ganglia layers, branching off into collateral endings in myenteric ganglia and circular muscle (Spencer et al. 2020b), as well as in the submucosa (Spencer et al. 2020a).

To the best of our knowledge, vagal afferent axons and nerve endings have not been identified in the mouse colon. In their study, Powley et al. (2011) used anterograde tracing from nodose ganglia in rats and mice to identify three types of vagal afferents in the proximal small intestine and gastric antrum of a cohort of nine mice: villus afferents, crypt afferents, and antral gland afferents.

After anterograde labeling, we found that vagal afferent axons were sparse by the mid colon and never detected as far as the distal 30 mm from the anal sphincter, consistent with vagal afferent innervation of the rat colon (Wang and Powley 2000). A recent study suggested that the mouse distal colon received prominent vagal afferents (Osman et al. 2023). In that study, retrograde tracing was used whereby the tracer is penetrated into the colon wall during repeated injections. We have been unable to control the spread of very small volumes of retrograde tracers injected into the distal colon, where we obtained non-specific labelling in nodose ganglia.

### How do EC cells communicate with vagal afferent nerve endings?

Recent studies have proposed that EEC make synaptic connections with vagal afferent endings in the mouse colon and communicate via fast synaptic transmission with vagal afferent endings, using glutamate as a neurotransmitter (Kaelberer et al. 2018). To test the validity of this notion, we rationalized that anterograde labelling from nodose ganglia should also label vagal afferent endings in the mucosa that also make synaptic connections with EC cells. Our data could not verify this hypothesis. Understanding the transduction of sensory stimuli from the gut lumen to primary afferent terminals is essential to interpret how changes in gut microbiota and metabolites affect the gut-brain axis.

The idea that EEC may communicate with sensory nerve endings was first conceived based on in vitro co-culturing studies of trigeminal neurons and enteroendocrine cells (Bohorquez et al. 2015). To date, we are unaware of any studies that have measured close contacts between extrinsic sensory nerve endings in mouse colonic mucosa and EEC, in situ. This is a serious weakness in the notion that EEC communicate directly via fast synaptic transmission with vagal afferent endings in the body. Indeed, studies have shown that in culture dishes, isolated sensory neurons can sprout in a direction toward sensory neurons (de Luca et al. 2015).

### Problems using sections of gut to determine distances between EC cells and nerve endings

Previous studies, using intestinal cross sections, have made claims about the distance between EC cells and nerve endings, but cross sections lack a 3D perspective. We overcame this limitation by using whole-mount full-thickness colon preparations and Imaris software, allowing for precise 3D measurements of the nearest distances between mucosally projecting vagal afferent endings and EC cells.

Lundberg and colleagues used electron microscopy to study the relationship between epithelial ECs and nerve terminals of any origin (sensory or motor) and concluded “no typical synaptic arrangements were observed…” although “…the minimal distance between the E.C. and the nerve bundles was 150 to 250nm” (Lundberg et al. 1978). It was not clear how many animals were or EC cells were studied, but “typical synaptic arrangements were searched for in the study…” “but not observed electron microscopically.” Indeed, in rat colon, electron microscopy identified long processes of EC cells that approach the bases of the epithelial cells. It was concluded that these cells do not receive innervation (Kuramoto et al. 2007). These investigators also concluded that serotonin released from the long process of EC cells acts in a paracrine fashion to epithelial cells. Also, work from Koo and colleagues concluded they “…could not find specific relationships between nerve fibres and the processes of colonic 5-HT cells” (Koo et al. 2021).

### Difficulties using antibodies to identify vagal afferent axons in the gut

There are no known antibodies that selectively label vagal afferent axons. For instance, CGRP antibodies cannot distinguish the origin of axons or endings, whether intrinsic or extrinsic to the gut wall, and dense labeling hinders tracking a single axon. We addressed these issues by injecting tracer into vagal nerve cell bodies, ensuring exclusive labeling of vagal afferents and enabling the tracking of individual axon trajectories.

### Rapid turnover of ECs in the colon

Recent work (Wei et al. 2021) shows EC cells in the mouse colon rapidly turnover, with half being replaced every 2 weeks. This suggests that if spinal and vagal afferent endings formed synaptic contacts with EC cells, the sensory nerve endings would need to frequently form new synapses to connect with newly developed EC cells. Our current and previous studies indicate that this hypothesis is unlikely for 5-HT-containing EC cells in the mouse colon. Our study did not focus on peptide YY (PYY) or cholecystokinin (CCK) expressing EECs. However, Berthoud and Patterson (1996) examined CCK immunoreactive EECs and vagal afferent nerve endings, finding that most vagal axons were tens to hundreds of microns away from the nearest CCK-IR cell and suggested that CCK acts on vagal fibers in a paracrine manner. Our findings are in line with the conclusions of Berthoud and Patterson.

## Conclusions

The findings suggest that any substances released by 5-HT-containing ECs are likely to act via diffusion onto mucosa-projecting vagal afferent nerve endings, which then relay sensory information to the brain.

## Data Availability

All data in this study is available on request from the corresponding author.
